# Psychological Distress During the COVID-19 Pandemic in Patients With Mental or Physical Diseases

**DOI:** 10.3389/fpsyg.2021.703488

**Published:** 2021-08-12

**Authors:** Claudia Oppenauer, Juliane Burghardt, Elmar Kaiser, Friedrich Riffer, Manuel Sprung

**Affiliations:** ^1^Division of Clinical Psychology, Department of Psychology and Psychodynamics, Karl Landsteiner University of Health Sciences, Krems, Austria; ^2^University Hospital for Psychosomatic Medicine Eggenburg, Psychosomatisches Zentrum Waldviertel, Eggenburg, Austria; ^3^Psychiatric Rehabilitation Clinic Gars am Kamp, Psychosomatisches Zentrum Waldviertel, Eggenburg, Austria

**Keywords:** COVID-19, pandemic, mental health, psychological distress, psychotherapy

## Abstract

The coronavirus disease 2019 (COVID-2019) and the consequences of the pandemic on individuals’ social, economic, and public lives are assumed to have major implications for the mental health of the general population but also for patients already diagnosed with psychological disorders. The aim of the present study was to investigate the psychological distress during the COVID-19 pandemic in patients with psychological disorders or physical health conditions in inpatient mental and physical treatment programs. A total 2710 patients completed COVID-19 related questions concerning their psychological distress and financial burden during the pandemic. Patients with psychological disorders reported the highest level of psychological distress and financial burden compared to patients with physical health conditions. Furthermore, most patients with psychological disorders attributed their individual psychological distress to the COVID-19 pandemic. In comparison to patients with physical health conditions, patients with psychological disorders are more strongly impacted by the COVID-19 pandemic and have an additional need for psychological/psychotherapeutic treatment due to the COVID-19 crisis. The findings stress the importance of continuous psychosocial support and availability of psychosocial support services for patients with psychological disorders during the pandemic.

## Introduction

In March 2020 the World Health Organization (WHO) declared the coronavirus disease 2019 (COVID-19) a pandemic and highlighted its implications on mental health and psychosocial well-being. Clearly, the pandemic can act as a major stressor similar to other well-known stressors such as stressful live events or traumas, which will according to the diathesis–stress model lead to psychological disorders among vulnerable individuals (Siddaway, 2020). In line with this, recent studies on the impact of the COVID-19 pandemic on mental health have demonstrated increased prevalence rates of generalized anxiety disorder, depressive symptoms, PTSD, and sleep disturbances among both the general population and patients with mental illness (Cao et al., 2020; Tull et al., 2020; Wang et al., 2020; Zhang et al., 2020). A Chinese study reported high prevalence rates of mental health problems following the COVID-19 outbreak: 25.5% symptoms of anxiety disorders, 16.9% depression, and 26.2% for insomnia in persons seeking professional psychiatry outpatient support. Additionally, 20.9% of patients diagnosed with psychological disorders reported deteriorating mental health and limited access to psychiatric care as a result of travel restrictions, isolation at home, and fear of coronavirus infection in hospitals (Zhou et al., 2020).

An Austrian online survey investigating the effects of the pandemic showed that 4 weeks into the lockdown prevalence rates of depression (21%) and anxiety symptoms (19%) were increased compared to epidemiological data before the lockdown. Further, the results of this study revealed that younger age (<35 years), female gender, unemployment, and low income are significant risk factors for mental distress during the COVID-19 lockdown (Pieh et al., 2020). Particularly younger individuals and healthcare workers are at a higher risk for depressive symptoms or poor sleep quality (Guessoum et al., 2020; Huang and Zhao, 2020). Further risk factors are presence of chronic/psychiatric illness, student status, and frequent exposure to social media/news related to COVID-19 (Xiong et al., 2020). Yao et al. (2020) suggested that alongside the COVID-19 pandemic there is a parallel epidemic of fear, anxiety, and depression since patients with mental disorders had restricted access to outpatient psychiatric care.

Furthermore, findings from US studies hypothesize that COVID-19-related negative emotional and social consequences are linked to past-month suicidal ideation and attempts in adults (Ammerman et al., 2020; Courtet et al., 2020; Reger et al., 2020). Besides research on the impact of the pandemic on mental health status and care, it is also hypothesized that psychiatric illness may be a potential risk factor for the COVID-19 disease (Taquet et al., 2020).

The negative effects of the COVID-19 pandemic on the economic situation and individuals’ financial well-being and financial worries (Tull et al., 2020) is well established (Asmundson and Taylor, 2020; Barrafrem et al., 2020; Goodell, 2020). Financial hardship caused by unemployment and decreasing revenues are likely involved in the psychosocial effects of the pandemic. There is plenty of evidence that a low socio-economic status is associated with mental disorders. Since, people with mental health issues have lower incomes, they are more likely to experience the consequences of the COVID-19 crisis.

Many European countries implemented severe restrictions on the social, economic, and public life for the entire populations. Austria was early to implement its first lockdown from March 13th to April 30th 2020. During this first lockdown all rehabilitation clinics in Austria, were closed and consequently the treatment of people with high burden of psychological disorders was severely limited.

The present study is part of a large-scale longitudinal research project investigating the consequences of the COVID-19 pandemic for people with mental and physical diseases in Austria. In the present study we investigated the psychological impact of the COVID-19 pandemic on patients in mental and physical inpatient treatment programs after the first lockdown.

## Materials and Methods

### Participants and Procedure

Data was collected from seven different rehabilitation/treatment clinics in Austria in an observational and cross-sectional study design. The clinics are specialized on the treatment of orthopedic, neurologic, oncologic, pulmonological, and psychosomatic/psychiatric patients. Patients answered the relevant questions at the beginning of their treatment from June to October 2020. For the psychiatric patients COVID-19 related questions were added to the psychological routine outcome monitoring relying on electronically assessed patient reported outcome data (Holzner et al., 2012). All inpatients of the seven clinics were included in the study despite of poor German language skills or if patients refused to answer the COVID-19 questionnaire.

We measured participants’ distress and financial burden during the pandemic as well as their subjective perception of a relationship between their mental health burden and the COVID-19 crisis. The final sample consisted of 2710 adults. Age ranged from 18 to 97 years (*M*_age_ = 58.0, SD = 14.14). The majority of patients (87.6%) were recruited from clinics with the focus on rehabilitation of physical diseases: 384 (14.2%) patients with neurological disorders, 1378 (50.8%) patients with orthopedic disorders, 135 (5.0%) patients with pulmonary diseases, 477 (17.6%) cancer patients, and 336 (12.4%) patients with psychological disorders.

The descriptive data analysis was conducted with the SPSS (version 26).

### Materials

#### Psychological Distress

All patients answered four items on perceived psychological distress from the emotional functional (EF) scale of the quality of life questionnaire for oncology patients, EORTC QLQ-C30 (Aaronson et al., 1993). The EF scale is recommended for screenings for psychological distress (Andersen et al., 2014). The functional scales of the EORTC QLQ-C30 are linearly transformed in scores ranging from 0 to 100, with higher scores representing higher (= better) levels of functioning. Psychometric studies about the EORTC QLQ-C30 recommend to use a cut-off point <66.7 to identify patients with psychological distress levels relevant for psychological support (Aaronson et al., 1993). Further, the financial difficulties item from the EORTC QLQ-C30 was adapted to relate to the COVID-19 crisis. Participants rated their levels of distress on a 4-point scale from 1 = “not at all” to 4 = “very much.”

#### Subjectively Experienced Impact of the COVID-19 Crisis on Mental Health

Five items assessed the subjectively perceived connection between psychological distress and the COVID-19 pandemic. Patients rated whether psychological distress symptoms were related to the current COVID-19 pandemic on a 4-point scale (1 = “not at all” to 4 = “very much”) and whether the psychological distress symptoms had changed positively or negatively due to the pandemic. Finally, patients reported whether they experienced a need for psychological/psychotherapeutic treatment due to the COVID-19 pandemic (Table 1).

**TABLE 1:** Questions regarding the relationship between psychological distress and the COVID-19 pandemic.

**Issue**	**Items**
Subjectively perceived relationship between the COVID-19 pandemic and psychological distress	(1) How much do you attribute these complaints to the COVID-19 pandemic?^1^
	(2) How much have these complaints changed since the COVID 19 pandemic?^1^
	(3) Was this change a(n):
	(a) Improvement (b) Deterioration
Psychological support/psychotherapeutic treatment needs	(4) Do you see a need for psychological/psychotherapeutic treatment in connection with the condition leading to admission?^2^
	(5) Do you see a need for psychological psychotherapy treatment due to the COVID-19 pandemic?^2^

*^1^Response format: (0) not at all to (3) very much. ^2^Response format: yes/no.*

## Results

Patients with mental illness reported the highest level of psychological distress (*M* = 39.06, SD = 21.30) followed by neurologically ill patients. Neither group reached the sum score for sufficient emotional functionality (≥66.7). Among patients with physical diseases the orthopedic patients showed the lowest level of psychological distress. The other patient groups had comparably low levels of psychological distress. Further, patients with mental illness reported the highest level of financial difficulties (*M* = 36.32, SD = 32.49) compared to patients with physical health conditions (*M* = 13.09, SD = 23.78). Table 2 displays psychological distress and financial difficulties for the different patient groups.

**TABLE 2:** Psychological distress (EF scale) in different patient groups.

**Group**	**Psychological distress (*M*/SD)**	**Financial burden (*M*/SD)**
Psychiatry (*N* = 336)	39.06 (21.30)	36.32 (32.49)
Neurology (*N* = 384)	64.13 (20.05)	18.40 (26.66)
Orthopedic (*N* = 1378)	69.04 (19.59)	16.33 (25.13)
Pulmonology (*N* = 135)	61.48 (20.23)	13.09 (23.78)
Oncology (*N* = 477)	63.00 (19.01)	20.75 (27.84)

**FIGURE 1:**
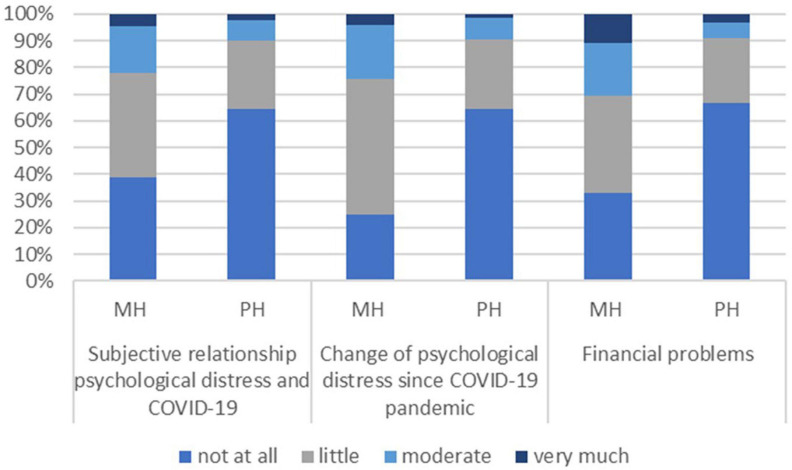
Psychological distress and financial problems during the COVID-19 pandemic in patients with mental health (MH) and physical health conditions (PH).

Overall, the majority of patients with physical diseases (64.6%) perceived no connection between their psychological distress level and the COVID-19 pandemic (Figure 1).

Patients with physical diseases also experienced no changes in their psychological distress in response to the COVID-19 pandemic (64.2%). In contrast, patients with mental illness perceived a connection between their level of psychological distress and the COVID-19 pandemic: 61.1% attributed their distress slightly or strongly to the crisis and 75.2% reported slight to strong psychological changes during the pandemic. In both groups the perceived psychological distress due to the COVID-19 pandemic was more likely to worsen than to improve: 77.2% of mental illness patients and 66.3% of physical disease patients had perceived a deterioration due to the COVID-19 pandemic (Table 3).

**TABLE 3:** Subjective psychological distress connected with the COVID-19 crisis and financial difficulties in patients with physical and mental diseases.

**Psychosocial distress and COVID-19**	**Response format**	**All patients (%)**	**Patients with mental illness (%)**	**Patients with physical disease (%)**
Attribution of psychosocial distress and the COVID-19 pandemic	Not at all	61.4	39.0	64.6
	Little	27.0	39.0	25.3
	Moderate	9.1	17.6	7.95
	Very much	2.5	4.5	2.19
Change of psychosocial distress since COVID-19 pandemic	Not at all	59.4	24.8	64.2
	Little	29.2	50.7	26.1
	Moderate	9.6	20.3	8.0
	Very much	1.9	4.2	1.6
Psychosocial distress change	Improved	32.0	22.8	33.7
	Worsened	68.0	77.2	66.3
Financial difficulties	Not at all	63.1	32.9	66.8
	Little	25.4	36.5	24.0
	Moderate	7.6	19.9	6.0
	Very much	4.0	10.7	3.2

Concerning patients’ financial situation, the majority of patients with physical diseases (66.8%) reported no financial problems related to the pandemic. Whereas 67.1% of patients with mental illness reported some kind of financial problems in relation to the COVID-19 crisis.

The results also demonstrate that mentally ill people report an additional need for psychological/psychotherapeutic treatment due to the COVID-19 pandemic (24.9%). Other patient groups with physical diseases, e.g., pulmonological patients report a higher need for psychological/psychotherapeutic support due to their health problems (33.3%) instead of in response to the COVID-19 crisis (Table 4).

**TABLE 4:** Need for psychological/psychotherapeutic support.

**Patient group**	**Need for psychological interventions *N* (%)**	**Need for psychological interventions/COVID *N* (%)**
Psychiatry (*N* = 336)	–	83 (24.9)
Neurology (*N* = 384)	140 (36.5)	33 (8.6)
Orthopedic (*N* = 1378)	237 (17.2)	69 (5.0)
Pulmonology (*N* = 135)	45 (33.3)	14 (10.4)
Oncology (*N* = 477)	Missing	34 (7.1)

## Discussion

This study evaluated the psychological impact of the COVID-19 pandemic on patients with different physical or mental diseases, during rehabilitative inpatient treatment. In contrast to patients with physical illness, patients with mental illness reported a high level of psychological distress due to the COVID-19 pandemic and also expressed an additional need for psychological/psychotherapeutic treatment due to the COVID-19 pandemic. A recent review and meta-analysis indicated that studies about the mental health consequences of the COVID-19 lockdowns are very heterogenous and the general psychological impact was found to be very small for the healthy population indicating that the majority of investigated individuals were resilient to the effects of the crisis and only small effect sizes could be found for depression and anxiety. Other potential psychosocial dimensions such as social support, loneliness, general distress, negative affect, and suicide risk were not significant. Further gender and age had no impact (Prati and Mancini, 2021).

Irrespective of mental health consequences of the COVID-19 pandemic on the healthy population, patients with serious psychological disorders perceived significant psychological distress in relation to the pandemic. Italian patients were four times more affected by pandemic related stress, anxiety, and depressive symptoms than a non-psychiatric control group. Mental health implications are also found in the general population for women and younger individuals (Rossi et al., 2020).

Patients with psychological disorders further report increased financial hardship. This group of individuals with severe mental health problems typically suffer from high unemployment rates even without a pandemic related crisis. It seems likely that many patients experience substantial everyday life limitations due to their limited financial resources, which creates the risk to suffer from financial straits. Since major parts of psychotherapeutic treatment in Austria are self-financed (Riffer et al., 2018), limited financial resources are relevant barriers for psychological and psychotherapeutic support.

These findings highlight an increased need for psychological care among patients with mental illness during the COVID-19 pandemic (Iasevoli et al., 2020; Titov et al., 2020). Further, to face the long-term consequences of the pandemic, access to psychotherapeutic care is important for all patients with mental or physical diseases, in order to prevent the development of chronical psychological distress, anxiety, and mood disorders (Dawel et al., 2020). Besides the mental health impact of the COVID-19 pandemic, patients with severe mental illness have a two to three times higher mortality rate than the general population due to unhealthy life style behaviors like smoking or obesity which are related to cardiovascular diseases, type 2 diabetes, and respiratory tract diseases like COPD. Approximately 14.3% of deaths worldwide are related to mental disorders – thus, mental disorders are one of the most considerable causes of deaths worldwide (Walker et al., 2015). Furthermore, these physical health conditions are well known risk factors for a severe COVID-19 disease course (Williamson et al., 2020). Several studies reported a relationship between mental disorders and an increased risk for SARS-CoV-2 infection and associated hospital duration, morbidity, and mortality (Li et al., 2020; Taquet et al., 2020).

The results of the present study show, that patients with mental illness are strongly impacted by the COVID-19 pandemic, much more than people with physical illness. It is therefore crucial that psychological/psychotherapeutic treatment, including inpatient treatment for people with mental illness is available throughout the current pandemic. Besides, data from the present study also highlight the need for psychological/psychotherapeutic support among patients with physical illness. Particularly for pain and cancer patients, the relevance and positive impact of psychological interventions have been widely established (Mehnert and Koch, 2008; Williams et al., 2012; Hughes et al., 2014; Bradford and Chan, 2017).

Even though the results are relevant for public health strategies during future pandemics lockdowns and other restrictions the study has limitations. The results are restricted to a specific patient population and are based on self-reported measures. It is unclear whether the results will generalize to outpatients with chronical physical health problems or mental illness who are not in need of a stationary stay or are still waiting for clinic admission. Further the patients’ retrospective evaluation of the impact of COVID-19 on subjective psychosocial distress could be affected by conditions not related to COVID-19 for instance poorer physical health or financial resources linked to factors we did not investigate.

## Conclusion

The study results emphasize the importance of monitoring the psychological impact of the COVID-19 pandemic on patients with mental or physical illness. From a public health perspective access and supply of sufficient psychological/psychotherapeutic services will be crucial to counter the negative consequences of pandemics (Leonardi et al., 2020) and to offer low-threshold psychological support like telephone hotlines. Moreover, it will be important to strengthen the patient–therapist/psychiatrist relationship in order to prevent a mental health crisis among patients and provide appropriate psychotherapeutic or pharmacological interventions to manage stress and disease related symptoms (Shinn and Viron, 2020). Since in Austria psychological support or psychotherapy must be financed primarily by the patients themselves, our study also highlights the urgent need for financing models, which allow an easy to afford access for people with psychological problems.

Future studies should investigate the long-term effects of the COVID-19 crisis in longitudinal designs and control for other physical or mental health related factors with a possible impact on psychosocial distress.

Further research is planned to evaluate changes on specific symptoms and diagnoses like anxiety, depression, or somatic symptoms during the COVID-19 pandemic as well as psychological risk and protecting factors in patients with mental or physical illnesses.

## Data Availability Statement

The datasets presented in this article are not readily available because of the vulnerability of the study sample. Participants of this study did not agree for their data to be shared publicly, so supporting data is not available. Requests to access the datasets should be directed to CO, claudia.oppenauer@kl.ac.at.

## Ethics Statement

The studies involving human participants were reviewed and approved by Karl Landsteiner University Ethics Commission. The patients/participants provided their written informed consent to participate in this study.

## Author Contributions

CO, JB, and MS: conceptualization. CO and JB: methodology, formal analysis, investigation, data curation, and writing-original draft preparation. MS, FR, and EK: writing-review and editing. All authors have read and agreed to the published version of the manuscript.

## Conflict of Interest

MS is the scientific director of the University Clinic for Psychosomatic Medicine Eggenburg, PSZW (Eggenburg and Gars). FR is the medical director of the University Hospital Eggenburg. EK is the chief physician of the University Hospital Eggenburg. The remaining authors declare that the research was conducted in the absence of any commercial or financial relationships that could be construed as a potential conflict of interest.

## Publisher’s Note

All claims expressed in this article are solely those of the authors and do not necessarily represent those of their affiliated organizations, or those of the publisher, the editors and the reviewers. Any product that may be evaluated in this article, or claim that may be made by its manufacturer, is not guaranteed or endorsed by the publisher.
